# Soil Viral Communities Vary Temporally and along a Land Use Transect as Revealed by Virus-Like Particle Counting and a Modified Community Fingerprinting Approach (fRAPD)

**DOI:** 10.3389/fmicb.2017.01975

**Published:** 2017-10-10

**Authors:** Anja Narr, Ali Nawaz, Lukas Y. Wick, Hauke Harms, Antonis Chatzinotas

**Affiliations:** ^1^Department of Environmental Microbiology, Helmholtz Centre for Environmental Research—UFZ, Leipzig, Germany; ^2^Department of Soil Ecology, Helmholtz Centre for Environmental Research—UFZ, Halle/Saale, Germany; ^3^German Centre for Integrative Biodiversity Research (iDiv) Halle-Jena-Leipzig, Leipzig, Germany

**Keywords:** time-series analysis, virus, phage, community dynamics, virus-like particles, VLP, RAPD, soil

## Abstract

Environmental surveys on soil viruses are still rare and mostly anecdotal, i. e., they mostly report on viruses at one location or for only a few sampling dates. Detailed time-series analysis with multiple samples can reveal the spatio-temporal dynamics of viral communities and provide important input as to how viruses interact with their potential hosts and the environment. Such surveys, however, require fast, easy-to-apply and reliable methods. In the present study we surveyed monthly across 13 months the abundance of virus-like particles (VLP) and the structure of the viral communities in soils along a land use transect (i.e., forest, pasture, and cropland). We evaluated 32 procedures to extract VLP from soil using different buffers and mechanical methods. The most efficient extraction was achieved with 1× saline magnesium buffer in combination with 20 min vortexing. For community structure analysis we developed an optimized fingerprinting approach (fluorescent RAPD-PCR; fRAPD) by combining RAPD-PCR with fluorescently labeled primers in order to size the obtained fragments on a capillary sequencing machine. With the concomitantly collected data of soil specific factors and weather data, we were able to find correlations of viral abundance and community structure with environmental variables and sampling site. More specifically, we found that soil specific factors such as pH and total nitrogen content played a significant role in shaping both soil viral abundance and community structure. The fRAPD analysis revealed high temporal changes and clustered the viral communities according to sampling sites. In particular we observed that temperature and rainfall shaped soil viral communities in non-forest sites. In summary our findings suggest that sampling site was a key factor for shaping the abundance and community structure of soil viruses, and when site vegetation was reduced, temperature and rainfall were also important factors.

## Introduction

The first description of abundant virus-like particles (VLP) in environmental samples in the late 80s of the last century has substantially changed our view on the distribution and ecological relevance of viruses (Bergh et al., 1989). Since then many studies have shown the importance of viruses for their hosts' mortality and community composition (Weinbauer and Suttle, 1996; Danovaro et al., 2008; Shapiro et al., 2010; Payet and Suttle, 2013), the genetic landscape (Anderson et al., 2011), and the nutrient turnover (Wilhelm and Suttle, 1999; Suttle, 2005) in ecosystems. Although viruses have been found in most environments (Paez-Espino et al., 2016), many ecosystems are still grossly undersampled which keeps us from fully understanding the stability and dynamics of viral communities in a spatio-temporal context.

The temporal dynamics of viruses have so far been mainly investigated in marine and freshwater systems (Larsen et al., 2001; Laybourn-Parry et al., 2007; Needham et al., 2013). For instance, the abundance of free viruses in marine systems follows a seasonal cycle, with overall peak abundances in summer and fall (Jiang and Paul, 1994; Sandaa and Larsen, 2006; Parsons et al., 2012; Payet and Suttle, 2013). Viral communities also show seasonal dynamics which could be linked to changes in the community structure or abundance of their potential hosts (Wommack et al., 1999; Sandaa and Larsen, 2006; Helton and Wommack, 2009; Hurwitz et al., 2014). On the contrary, the temporal variability of soil viruses has only sporadically been addressed with the exception of studies with a rather epidemiological or agronomic focus (El-Didamony and Abdel-Fattah, 1998; Fuxa et al., 2001).

Virus abundances in soil have been reported to range between 10^7^ and 10^9^ virus like particles per g of soil (Williamson et al., 2003, 2005; Swanson et al., 2009; Srinivasiah et al., 2015) and exceed in some cases (e.g., Antarctic soils) the abundance of co-occurring bacteria by up to 8,200-fold (Williamson et al., 2007). So far it is unclear, if, to which degree, and how environmental conditions affect viral numbers and viral community composition in soils. Fertilizer applications to soil have been shown to increase virus abundance (Chen et al., 2014; Doan et al., 2014), and changes influencing the bacterial community in general also affect the abundance of viruses and the diversity of viral communities (Srinivasiah et al., 2013, 2015; Doan et al., 2014). Viruses from forest soils are both more abundant and more diverse than viral communities from agricultural soils (Williamson et al., 2005), while different land use practices generally result in differing communities (Fierer et al., 2007; Srinivasiah et al., 2013). Still, time-series recording the temporal variability in different terrestrial ecosystems are needed to address questions regarding the temporal dynamics, the stability and responses of viral communities to changing environmental conditions.

One reason for the slower progress in soil viral ecology as compared to other fields of microbial ecology is the challenge to establish appropriate low-cost methods for a reproducible monitoring of viral abundances and community compositions. Usually, an extraction step is required to collect viral particles from soils. There have been different extraction buffers described which are usually combined with some form of mechanical treatment, like vortexing (Williamson et al., 2003; Wommack et al., 2009), sonication (Kim et al., 2008; Wommack et al., 2009), bead-beating (Williamson et al., 2013), or shaking (Casas and Rohwer, 2007; Fierer et al., 2007) followed by a centrifugation step to separate the soil particles, bacteria and viruses. Previous studies showed that extraction efficiencies mainly depend on soil properties and soil types (Trubl et al., 2016). Thus, it is reasonable to assume that the extraction protocol needs to be evaluated for each soil or habitat, in order to allow reproducible results for monitoring of viral abundances.

While most of the studies on the structure of viral communities in different environments were conducted using metagenomic approaches (e.g., Desnues et al., 2008; Adriaenssens et al., 2015; Brum et al., 2015), low-cost rapid community-level fingerprinting methods have rarely been successfully applied (Schroeder et al., 2003; Zhong et al., 2014). Fingerprinting methods have the advantage of faster analysis and higher sample throughput in routine screening surveys, albeit at the cost of less detailed data compared to sequencing approaches (van Dorst et al., 2014). A particular challenge is the lack of shared viral marker genes. There have been attempts to use the genes for polymerases, capsid proteins or other structural proteins as marker genes for certain viral groups (reviewed in Adriaenssens and Cowan, 2014). However, only a small range of usually related viruses contains these genes, which are thus limited in their use as marker genes. When surveying the whole viral community, a less targeted approach needs to be used.

The most common fingerprinting technique currently used for soil virus assemblages is RAPD-PCR (randomly amplified polymorphic DNA-PCR; Williams et al., 1990; Hadrys et al., 1992; Srinivasiah et al., 2013, 2015). This approach is based on the use of one primer of around 10 nucleotides length, which binds several times in the genome, resulting in banding patterns that can be analyzed. Unfortunately, the gel-based analysis of the produced fragments is time consuming and heavily dependent on the experience of the experimenter. Additionally, it has been reported that replicate samples run on two different gels can differ to up to 20% (Winget and Wommack, 2008; Helton and Wommack, 2009).

In the present study we performed a 1 year time-series analysis of soil viral communities along a transect with different land use practices (i.e., forest, pasture, cropland) in the Hainich National Park (Thuringia, Germany). For this purpose we developed a modified RAPD-PCR technique for community structure analysis, called fRAPD (i.e., fluorescent RAPD), by using fluorescence labeled primers in order to size the fragments on a capillary sequencing machine. This fRAPD approach and an optimized VLP extraction protocol were then used to answer the following questions: (i) Do the changes in the viral (i.e., VLP) abundance over 1 year follow similar patterns along the transect? (ii) Does the composition of the viral community change over time and differ along the transect? (iii) Which climatic and soil specific factors are relevant for changes in the viral abundance and community structure?

## Materials and methods

Sampling was conducted at the Hainich Critical Zone Exploratory (Hainich CZE) in Thuringia, Germany, within the framework of the DFG funded AquaDiva project (CRC 1076). A detailed description of the study site is provided by Küsel et al. (2016). Briefly, the Hainich National Park is the largest connected deciduous forest in Germany. The Hainich CZE was established at the eastern slope of Hainich range, which has a mean inclination of ~2°. The underlying geological units building the soil bedrocks belong to the Middle and Upper Muschelkalk (mainly marlstones, limestones) and Lower Keuper (clay, siltstones). The soil sampling sites were taken from a transect oriented in W-E direction and covers the land use types forest, pasture and cropland (Küsel et al., 2016).

### Field sampling

**Figure 1:**
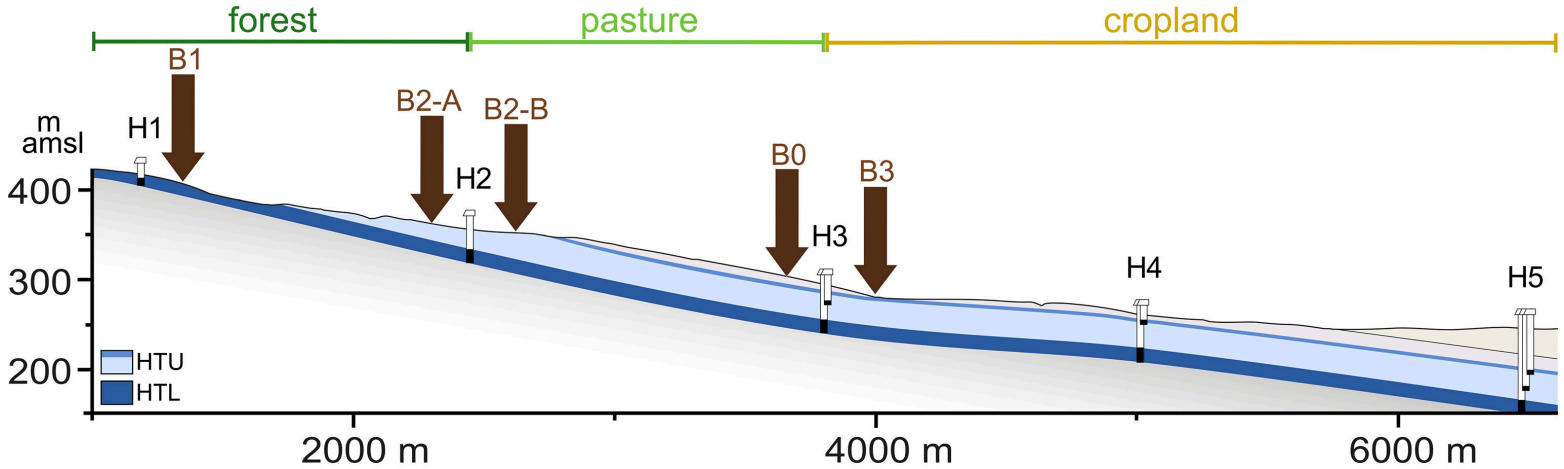
Sampling locations at the eastern Hainich hillslope. The cross section (vertically 4x exaggerated) shows the Upper Muschelkalk aquifer assemblages: HTU, Hainich transect upper aquifer assemblage; HTL, Hainich transect lower aquifer assemblage; H1 to H5, groundwater monitoring wells; B0, soil sampling location for the extraction efficiency assessment; B1–B3, locations for regular soil sampling; m amsl, meter above mean sea level. Adapted base figure from Küsel et al. (2016).

For the investigation of extraction efficiencies, a large topsoil sample (~1 kg, 1–15 cm depth, rendzic leptosol) was taken at location B0 (pasture, at N51° 06.654′, E010° 25.383′, see Figure 1) in June 2014, sieved to 4 mm removing litter, roots and stones and stored at 4°C until further processing as described below.

From August 2014 to August 2015 soil samples were taken every 4 weeks at two forest sites (B1 at N51° 06.207′, E010° 23.844′, and B2-A at N51° 06.507′, E010° 24.702′), one pasture site (B2-B at N51° 06.552′, E010° 24.859′), and one cropland site (B3 at N51° 06.653′, E010° 25.325′). All soils were categorized as rendzic leptosols, except for B2-A, which is a brown calcareous soil. All sampling locations were close to the previously established groundwater monitoring wells within the Hainich CZE (see Figure 1). Soil sampling campaigns in December 2014 and January 2015 were skipped due to unfavorable weather conditions. For each soil sample the upper 10 cm were collected with a soil driller and sieved to 4 mm to remove litter, roots and stones. At each site, five replicates within an area of 1.5 × 2 m (minimal distance of two replicates was 64 cm, maximal distance 189 cm) were sampled and stored at 4°C for a maximum of 2 days until further processing.

### Extraction of virus-like particles (VLP)

To evaluate the most efficient method to extract virus like particles (VLP) from the rendzic leptosol soil, the efficiency of 32 combinations of several commonly used buffers with mechanical treatments was tested in a single extraction without replicates. Between 6.5 and 7.5 g of soil was weighed into 50-mL polypropylene tubes. To each tube 15 mL of either 1 × or 0.1 × buffer [potassium citrate (PC; per liter 1.44 g Na_2_HPO_4_^*^7 H_2_O, 0.24 g KHPO_4_; pH 7.0), sodium pyrophosphate (SP; 10 mM; pH 7.0), phosphate buffered saline (PBS; 1 ×; pH 7.4), or saline magnesium (SM; per liter 5.8 g NaCl, 2 g MgSO_4_^*^7 H_2_O, 50 mL 1 M Tris-HCl (pH 7.5)] were added. One of the following mechanical methods were used: vortexing (20 min at maximum intensity), shaking (10 min vortexing at low intensity), sonication (three times 1 min at 100 W, 47 kHz with 30 s breaks of incubation on ice), or bead-beating (~20 glass beads with 5 mm diameter added, 1 min vortexing at maximum intensity, followed by 10 min shaking on an orbital shaker at 200 rpm). Samples were centrifuged at 3,000× g for 30 min at 4°C and the supernatant was purified from bacteria and other large particles using 0.45 and 0.22-μm syringe filters (cellulose acetate, Lab Logistics Group GmbH, Meckenheim, Germany). The viral concentrate was stored in sterile glass vials with a drop of chloroform at 4°C.

For the extraction of the soil samples from the monthly sampling campaigns we used the 1 × SM buffer in combination with vortexing as described above. The VLP from the five replicates taken from each site were extracted individually and all following steps were done for each replicate sample separately.

### Microscopic quantification of VLPs

For enumeration of virus like particles (VLP), 3 mL of sterile water mixed with up to 100 μL viral concentrate was filtrated on a 0.02-μm Anodisc aluminum oxide filter (27 mm diameter, Whatman, GE Healthcare, Kent, UK) with a 0.45-μm supportive filter (cellulose nitrate, Sartorius Stedim Biotech GmbH, Göttingen, Germany). Anodisc filters were dried and stained with 2× SYBR Gold (Invitrogen, Thermo Fisher Scientific, Waltham, USA) according to the protocol of Thurber et al. (2009). At least 20 images of every sample were taken automatically using an Axio Observer.Z1 microscope (Zeiss, Jena, Germany) at 1,000× magnification as described by Tischer et al. (2012). The images were then analyzed with the ACMEtool2 (Version 2012-07-15, http://www.technobiology.ch/index.php?id=acmetool), counting all particles with the following parameters: Area > 15 and Area < 80 and SBR > 1 and Circularity > 0.6. These parameters were experimentally determined to capture as many VLP as possible while excluding bacterial cells. The number of VLPs per microscopy image was calculated based on soil dry weight.

### DNA-extraction

Soil samples were prepared as described in section Extraction of Virus-Like Particles (VLP), and the 0.2-μm-filtrated supernatant of the soil slurry was used for additional processing steps by ultracentrifugation. Using a high speed centrifuge (Optima XPN-80 Ultracentrifuge with rotor SW 32 Ti, Beckman Coulter, Krefeld, Germany; each run: 22,000 rpm for 2 h at 4°C) the viral particles were collected in centrifugation tubes and resuspended in 500 μL TM-buffer (50 mM Tris-HCl, 10 mM MgSO_4_, 0.02-μm-filtrated) overnight at 4°C. The viral concentrates were stored for up to 1 week in 30 μL chloroform. Then samples were DNase treated to remove free DNA. To each 500 μl sample of ~1 μL Ambion® DNase and 50 μL 10× buffer (Thermo Fisher Scientific, Waltham, USA) were added and incubated overnight at 30°C. As a control of DNA removal the linearized plasmid pCR 2.1 (for production of the plasmid see Supplementary Experimental Procedures) was added to the sample before DNase treatment. After each treatment the sample was screened for the plasmid using M13 PCR primers (PCR conditions are available in Supplementary Experimental Procedures). Between repeated DNase treatments the VLPs were purified from proteins with a chloroform extraction. An equal volume of chloroform was added and the sample inverted several times. The sample was centrifuged at 3,000 rpm for 30 min at 4°C and the upper aqueous phase was transferred to a new tube for another DNase treatment. When the PCR for the reference plasmid was negative, the samples were considered free of residual non-encapsulated DNA and frozen at −80°C. DNA extraction was performed as described by Thurber et al. (2009), which combines a formamide/cetyltrimethylammonium bromide (CTAB) incubation, proteinase K treatment and a phenol/chloroform extraction step. DNA was quantified using the Qubit® dsDNA HS Assay Kit (life technologies, Thermo Fisher Scientific, Waltham, USA) following the manufacturer's instructions and adjusted to 1 ng/μl for all samples.

### Fluorescent RAPD (fRAPD)

A set of primers was evaluated for the production of banding patterns. Primers HDC-1 (5′-CGCCGCCGCC-3′), HCB-1 (5′-CCAGCAGCAG-3′), LWHS-1 (5′-GTTCGGGTCG-3′), RLWLS-1 (5′-GCGATCCACG-3′), AAWZS-1 (5′-CACCACCTGC-3′), SJT-1 (5′-GCGGCGCATC-3′), SKS-1 (5′-GGCGGCGGCG-3′), and SWI-2 (5′-AGCGGCGCAT-3′; Srinivasiah et al., 2013) were used for amplification of viral DNA obtained from environmental samples. Primer AAWZS-1 (5′-CACCACCTGC-3′) was selected as it produced the most consistent banding patterns. A pseudo-nested PCR was chosen, as the FAM-labeled primer performed rather poorly, probably due to sterical issues caused by the size of the label. The first PCR contained 2× HotStarTaq *Plus* Mastermix (Qiagen, Venlo, Netherlands), 10 pmol primer AAWZS-1 without FAM-label (biomers.net GmbH, Ulm, Germany), 1 ng DNA and water up to 10 μL. The following PCR contained 2× HotStarTaq *Plus* Mastermix, 50 pmol primer AAWZS-1 with FAM-label (biomers.net GmbH, Ulm, Germany), 2 μL PCR product from the previous reaction and water up to 25 μL. Cycling was conducted as follows for both reactions: 94°C for 10 min, 20 cycles of (i) 94°C for 30 s, (ii) 43°C for 3 min, and (iii) 72°C for 1 min, final elongation was at 72°C for 10 min followed by cool down to 8°C.

PCR products were checked on 1.8% agarose gels (LE Agarose, Biozym, Hessisch Oldendorf, Germany) cast with 1× TBE buffer [90 mM Tris(hydroxymethyl)aminomethane, 90 mM Boric acid, and 2 mM EDTA]. A 2-log DNA ladder (New England Biolabs, Ipswich, MA, USA) was used as size reference and the gels were run at 100 V in 0.5× TBE buffer for 90 min. Gels were stained with ethidium bromide and the banding pattern visualized with a transilluminator (ChemiGenius, Syngene, Cambridge, UK).

PCR positive samples were purified using the QIAquick PCR Purification Kit (Qiagen, Venlo, Netherlands) according to the manufacturer's instruction. The samples were eluted in 30 μL purified water. The concentration of the PCR products was measured using the Qubit system, precipitated using ethanol (see Supplementary Experimental Procedures) and resuspended in Hi-Di™-formamide (Applied Biosystems, Thermo Fisher Scientific, Waltham USA). Five nanograms of each PCR product and 0.5 μL of size standard [custom MapMarker with ROX-labeled sizing bands at 50, 75, 100, 125, 150, 200, 250, 300, 350, 400, 450, 500, 550, 600, 650, 700, 750, 800, 850, 900, 950, 1,000, 1,050, 1,100, 1,150, 1,200, 1,250, 1,300, 1,400, and 1,500 bp, (BioVentures Inc., Murfreesboro, TN, USA)] and Hi-Di™ -formamide to a total volume of 10 μL were used for fragment analysis in the ABI PRISM 3130xl Genetic Analyzer (Applied Biosystems, Thermo Fisher Scientific, Waltham USA). Samples were analyzed on a 36-cm capillary filled with POP7-polymer at a run voltage of 10 kV and 3,000 s run time.

Peak sizes were determined using Gene Mapper V3.7 software (Applied Biosystems) and peaks between 50 and 1,500 bp were normalized and binned using an algorithm that identifies true peaks based on the peak area, which needs to be five times above the standard deviation calculated over all peaks (Abdo et al., 2006).

In order to analyze how reliable and reproducible the fRAPD analysis with a capillary sequencing machine is, four randomly selected DNA extracts were used for fRAPD analysis. From each fRAPD-PCR sample we transferred four subsamples to separate wells which were subsequently analyzed with the capillary sequencer. This plate was analyzed again after 2 days.

### Environmental data

Supplementary weather data was gained from the German Meteorological Service (DWD, www.dwd.de/cdc). Data for rainfall and average air temperature were used from the closest weather station in Görmar (station ID 6305, geographic location at N 51.2065 and E 10.4948). The daily values were recalculated to the average (temperature) or sum (rainfall) of 5 days, 10 days, and 4 weeks before sampling day.

The gravimetric water content of the soil samples was analyzed by oven drying the samples at 105°C for ≥24 h or until constant weight. The pH was determined in duplicate, using a glass electrode (WTW pH electrode SenTix 41, Xylem Analytics Germany Sales GmbH & Co. KG, Weilheim, Germany) in the supernatant of soil suspensions prepared using 1:2.5 mixtures of soil and 0.01 M CaCl_2_.

Before chemical analyses, visible plant residues and stones were removed by hand from the air-dried soil samples, and the samples were finely grounded to a size of 100 μm. Total carbon and total nitrogen contents of the soil samples were determined by dry combustion of triplicate sub-samples using a Vario El III C/H/N analyzer (Elementar, Hanau, Germany).

### Statistical analysis

Statistical analysis of the VLP counts was conducted using R version 3.2.3 (R Core Team, 2015). Spearman correlations were calculated for all environmental factors using the cor.test function of R. As the soil specific parameters (e.g., pH) were quite different for the sampling sites, we analyzed the samples for each sampling site individually. Multiple regression was conducted using R's glm function with Gaussian error distribution and log-link function. Predictors for the model were selected based on Spearman correlations. The performance of the models was estimated using the AIC (Sakamoto et al., 1986). The VIF (variance inflation factor) was calculated using vif function from the car-package (Fox and Weisberg, 2011). Adjusted *R*^2^ was calculated as follows:

$$\begin{array}{l} adj\;R2=1-\frac{\frac{\text{residual variance}}{\text{null variance}}*\left(sample\;size-1\right)}{residual\;degrees\;of\;freedom} \end{array}$$

The statistical analysis of the fRAPD data was conducted using the package vegan in R (Oksanen et al., 2016). For calculation of the percent similarity of replicate samples the Renkonen-index was used (Renkonen, 1938). To find out which parameters show significant correlations to the viral community composition, PerMANOVA was conducted with the adonis function in R (Anderson, 2001), Jaccards dissimilarity index (Jaccard, 1912), and permutation tests with 1,000 permutations. Using the metaMDS function nonmetric multidimensional scaling (Faith et al., 1987; Minchin, 1987) was used to visualize the similarity of the viral communities using 10,000 permutations. To determine the dissimilarity of the viral communities, Jaccard index was calculated using the vegdist function from the vegan package.

## Results

### VLP extraction efficiencies

**Figure 2:**
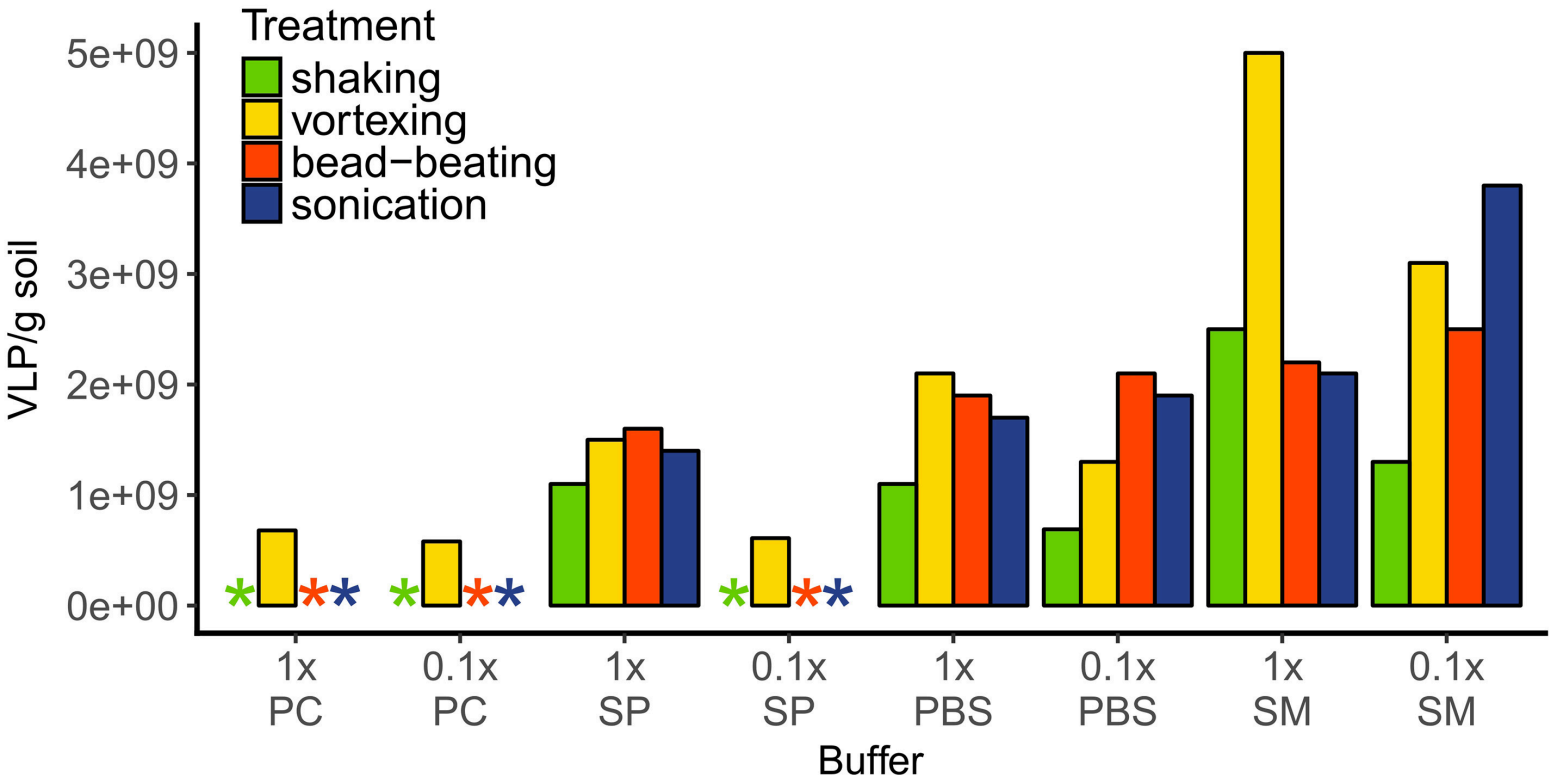
VLP extraction efficiencies of different buffer-mechanical treatment combinations. Extracted VLP were quantified with epifluorescence microscopy after staining with SybrGold. Asterisks indicate either a too low amount of VLPs or too high background fluorescence for correct counting. Buffers: PC, potassium citrate; SP, sodium pyrophosphate; PBS, phosphate buffered saline; SM, saline magnesium.

The most efficient extraction of VLP was achieved with 1× saline magnesium buffer (SM buffer) in combination with vortexing (resulting in 5 × 10^9^ VLP g^−1^ soil, Figure 2), which was then used for all further samples. Using potassium citrate buffer (PC buffer, in both concentrations) or diluted sodium pyrophosphate buffer (SP buffer) resulted in one order of magnitude lower VLP abundances (5.8–6.8 × 10^8^ VLP g^−1^ soil) or made the enumeration of the VLP impossible due to high background fluorescence; these methods were thus considered unsuitable. The 1× SP buffer and both PBS concentrations showed approximately similar extraction efficiencies (average 1.4–1.7 × 10^9^ VLP g^−1^ soil). In contrast, extraction efficiencies using SM buffer varied considerably depending on the mechanical treatment. Combining 1× SM buffer with shaking, bead beating or sonication gave similar viral counts of 2.1 × 10^9^ to 2.5 × 10^9^ VLP g^−1^, while vortexing increased the viral counts to 5 × 10^9^ VLP g^−1^. The extraction with diluted SM buffer resulted in the highest counts with sonication (3.8 × 10^9^ VLP g^−1^), intermediate counts with vortexing and bead-beating (between 2.5× and 3.1× 10^9^ VLP g^−1^), and lowest virus counts (1.3 × 10^9^ VLP g^−1^) with shaking.

### VLP abundance dynamics over 1 year

**Figure 3:**
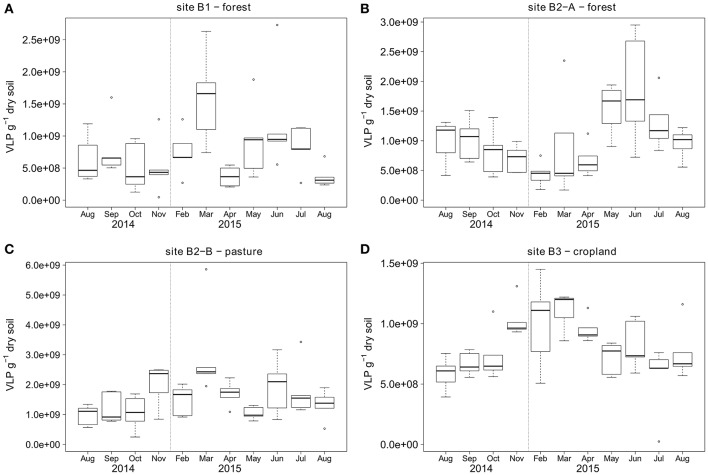
Temporal dynamics of VLP counts over the course of 1 year for sampling sites **(A)** B1 - forest, **(B)** B2-A - forest, **(C)** B2-B - pasture, **(D)** B3 - cropland. First and third quartiles around the median (bold line) are represented by the boxes and calculated based on five replicate samples. The dotted vertical line indicates the two omitted sampling campaigns in winter 2014/2015.

Topsoil VLP counts ranged from 2.4 × 10^7^ to 5.9 × 10^9^ VLP g^−1^ soil (dry weight) with a mean at 1 × 10^9^ VLP g^−1^ soil for the samples from two forest sites, one pasture, and one agricultural land use site during a 1 year sampling campaign (Figure 3). The forest soil at site B1 had the lowest overall VLP counts with a mean of 7.8 × 10^8^ VLP g^−1^, followed by cropland soil (site B3) and forest soil site B2-A with mean virus counts of 8.1 × 10^8^ and 1 × 10^9^ VLP g^−1^, respectively. Virus counts were highest at the pasture (site B2-B) with mean particle counts of 1.6 × 10^9^ VLP g^−1^. The standard deviation of the virus counts was lowest at site B3 (2.6 × 10^8^ VLP g^−1^), followed by sites B1 (5.7 × 10^8^ VLP g^−1^) and B2-A (6.1 × 10^8^ VLP g^−1^). The highest standard deviation was observed at site B2-B (8.9 × 10^8^ VLP g^−1^). Seasonal shifts were observed for all sites except B1 (see Figure 3). At the forest site B2-A, the virus counts were higher in summer (June until August) than in winter (February), whereas the opposite was observed for the pasture site B2-B and the cropland site B3.

### Factors influencing VLP abundance

**Figure 4:**
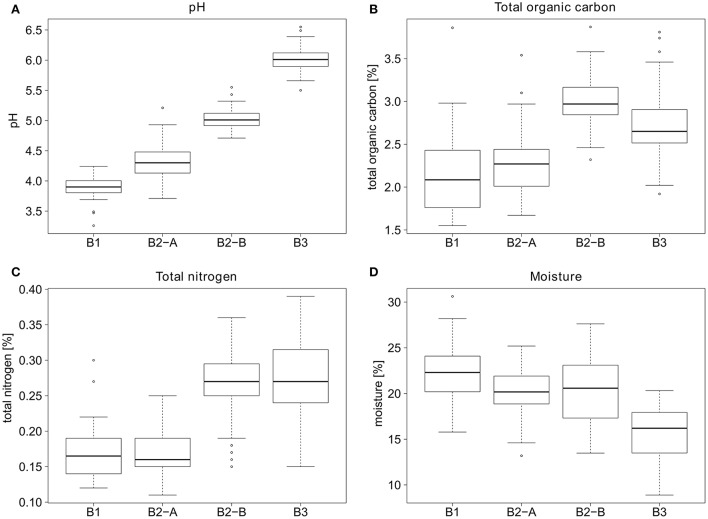
General parameters for the soil sampling sites. **(A)** pH values, **(B)** percent total organic carbon, **(C)** percent total nitrogen, and **(D)** percent moisture of the soil sampling sites.

Samples for VLP counts were taken from four different sites, which differed considerably in their soil parameters. The pH values ranged from 3.9 at site B1 (forest) to 6.0 at site B3 (cropland) (see Figure 4). Sites B1 and B2-A (both forest) had relatively similar organic carbon (2.1 and 2.3%) and organic nitrogen contents (0.167 and 0.170%). In comparison, sites B2-B and B3 had higher organic carbon (2.98 and 2.71%) as well as higher nitrogen contents (0.26 and 0.27%). The soil moisture was highest in B1 (forest) (22.3%), sites B2-A and B2-B had a slightly lower moisture content (20.2 and 20.5%) and B3 had the lowest overall soil moisture (15.7%).

Spearman correlations were used to evaluate the factors which might influence the soil VLP abundances. For the complete dataset (i.e., including all samples from all sites and months) a significant (*p* < 0.05) positive correlation of the VLP counts with soil pH, total nitrogen and total organic carbon was found (see Table 1). The strongest correlations were obtained with total nitrogen and total carbon (rho 0.33 and 0.38, respectively). A very weak but significant negative correlation was found for the carbon-to-nitrogen ratio (rho −0.14). Another very weak but significant negative correlation was found with sampling date (rho −0.14).

**Table 1:** Rank correlations of the VLP g^−1^ dry weight with environmental parameters using Spearman rho and associated *p*-values.

**Site**	**All**		**B1**		**B2-A**		**B2-B**		**B3**	
Land use			Forest		Forest		Pasture		Cropland	
No. samples	*n* = 217		*n* = 54		*n* = 53		*n* = 55		*n* = 55	
	**Rho**	***p***	**Rho**	***p***	**Rho**	***p***	**Rho**	***p***	**Rho**	***p***
Date	−0.14	0.042	−0.29	0.031					−0.27	0.048
pH	0.18	0.009	0.24	0.081	0.29	0.044			−0.25	0.063
Moisture	0.13	0.051					0.35	0.008		
TN	0.33	<0.001					0.31	0.021	0.31	0.020
TOC	0.38	<0.001			0.30	0.027				
C:N ratio	−0.14	0.038					−0.36	0.006		
Temperature D					0.47	<0.001197	−0.28	0.038	−0.57	<0.001
Temperature 5 D	−0.13	0.059			0.36	0.009	−0.39	0.003	−0.57	<0.001
Temperature 10 D					0.41	0.002	−0.31	0.020	−0.57	<0.001
Temperature M					0.41	0.002	−0.33	0.013	−0.57	<0.001
Rainfall D	−0.19	0.005	−0.27	0.051			−0.48	<0.001	−0.56	<0.001
Rainfall 5 D									−0.28	0.036
Rainfall 10 D					0.35	0.010	−0.29	0.032	−0.46	<0.001
Rainfall M	−0.15	0.026	−0.31	0.022			−0.25	0.071	−0.40	0.002

*Rho-values with p > 0.1 were omitted, rho values with 0.1 > p > 0.05 are printed in gray. TN, total nitrogen; TOC, total organic carbon; C:N ratio, carbon to nitrogen ratio; D, sampling day; 5 D, 5 days before sampling; 10 D, 10 days before sampling; M, 4 weeks before sampling. Values 5 D, 10 D, and M were calculated based on the mean (temperature) or sum (rainfall) of the measurements taken on each of the 5 days, 10 days, and 4 weeks before sampling*.

When analyzing individual sites, significant positive correlations of the VLP counts with pH (rho 0.29), total organic carbon (rho 0.30) were detected for B2–A (forest). At B2-B (pasture) VLP counts positively correlated with soil moisture (rho 0.35) and total nitrogen (rho 0.31). A negative correlation was observed with the carbon-to-nitrogen ratio (rho −0.36). VLP counts at B3 (cropland) correlated positively with total nitrogen (rho 0.31) and negatively with soil pH (rho −0.25) and sampling date (rho −0.27). A significant negative correlation was found for sampling date (rho 0.29) at B1 (forest).

Since temperature is a good proxy for the change of seasons, we further analyzed the significance of the air temperature and rainfall to explain the seasonal dynamics of the VLP counts (see also Figure 3). As it was not clear how fast changes in temperature and rainfall would be transferred to the soil and the microbial communities, we calculated these values for several timeframes, including the measurement at sampling day and the mean (temperature) or sum (rainfall) of the measurements taken on each of the 5 days, 10 days, and 4 weeks before sampling, respectively. Spearman correlation of air temperature with the VLP counts revealed a positive correlation at B2-A for all timeframes (rho 0.36–0.47). The same analysis revealed a more pronounced negative correlation for the pasture site (B3) (rho −0.57). VLP at B2-B also negatively correlated with air temperature, but with lower correlation strength (rho −0.28 to −0.39). Overall, it had little influence which time frame we choose for the analysis as either all (daily value and averaged values over 5 days, 10 days, and 1 month) or none of the temperature data showed a significant correlation with the viral counts.

Rainfall also influenced the VLP abundances. Especially for the non-forest sites (B2-B and B3) where no trees shield the soil surface from rain, significant negative correlations were found. For sites B2-B and B3, the strongest negative correlations with VLP counts were found for the rainfall on the day of the sampling (rho −0.48 and −0.56). At forest site B2-A a weaker positive correlation was found for the accumulated rainfall 10 days before sampling (rho 0.35) and for forest site B1 there was a weak negative correlation for the accumulated rainfall 1 month before sampling (rho −0.31).

Stepwise multiple regression indicated that overall the changes in VLP counts are best explained by the content of organic carbon, soil moisture and rainfall on the day of sampling even though these factors only explained about 20% of variation in the VLP abundances (adj*R*^2^ = 0.224; for a comparison of null, full and final model see Supplementary Table 1). For sites B1 and B2-B no good model could be found. However, for site B2-A almost 40% (adj*R*^2^ = 0.39) of the VLP count variation can be explained with total nitrogen content, rainfall 10 days before sampling and pH. At site B3 almost 40% of variation in virus counts can be attributed to temperature on the day of sampling (adj*R*^2^ = 0.396). For a list of all models tested see Supplementary Table 1.

### Optimization and reproducibility of fRAPD analysis

We evaluated eight primers for the production of banding patterns for the RAPD-PCR analysis. Primer AAWZS-1 showed the most consistent banding patterns in terms of band intensity. This is important for the detection and the subsequent analysis of the fluorescently labeled fragments as fragments with very high abundance can lead to off-scale peaks, i.e., fluorescent signals with a too high intensity, which cannot be correctly measured.

To assess the reproducibility of our new fingerprinting technique, we analyzed replicates of the four randomly selected DNA extracts. The electropherograms of the replicates of each sample were visually indistinguishable (see Supplementary Figure 1). When calculating the similarity of the samples based on the Renkonen-index (Renkonen, 1938), we found that the mean similarity between replicates was around 97%. The minimum observed similarity between replicates was 94.3% for three of the four samples. However, in one sample we observed substantially lower values (mean at 90.1%, minimum at 71.5%), which is most likely due to the high number of fragments with a very low abundance. In some of the replicated measurements these low-abundance peaks fall below the calculation threshold during normalization and are thus excluded from further analysis.

### fRAPD fingerprinting of soil viral communities

**Figure 5:**
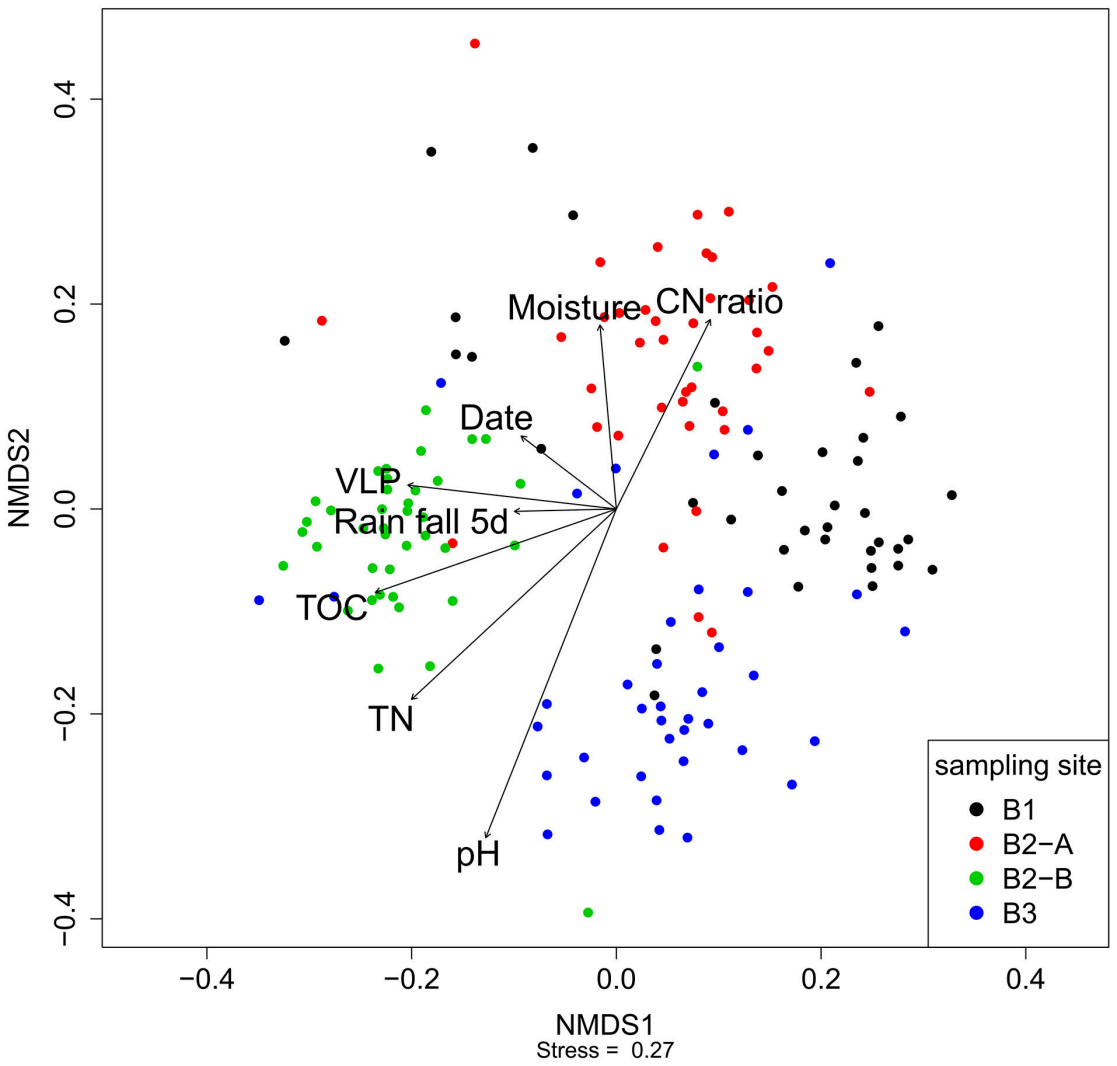
Nonmetric multidimensional scaling of the fRAPD profiles of the soil viral communities during a 1-year sampling campaign. Samples are color-coded based on sampling site. Arrows indicate significantly correlated environmental parameters. The length of the arrow indicates the effect size the parameter has on the community composition.

Nonmetric multidimensional scaling was used to visualize the similarity of the viral community fRAPD-fingerprints, and vectors for significantly correlated parameters were added. The samples clustered according to the sampling site (Figure 5). Factors affecting the community structure most were soil specific factors (pH, total nitrogen and organic carbon content, carbon-to-nitrogen ratio, moisture), but also the abundance of VLPs, the date and the total rainfall during the 5 days before sampling.

PerMANOVA-correlations of the environmental parameters with the complete viral community structure dataset found significant correlations for all soil specific parameters (pH, moisture, total nitrogen content, total organic carbon content, and carbon to nitrogen ratio), for sampling site, date, season of sampling, amount of VLPs and rainfall 5 days before sampling (Table 2). The *R*^2^-value indicates that the sampling site had the strongest influence on the composition of the viral community. For the individual sampling sites we found that total nitrogen content and sampling date had a significant influence on the viral community at B1 (forest), while at site B2-A (forest) sampling season and rainfall 5 days before sampling were significant factors shaping the community. Viral communities at B2-B (pasture) and B3 (cropland) were significantly correlated to soil moisture, sampling date, sampling season, air temperature, and rainfall. Additionally, there was a significant correlation of total nitrogen content to the viral community at site B3. Based on the *R*^2^-values, sampling season had the highest influence on the viral community at the individual sites.

**Table 2:** PerMANOVA correlations of the soil viral communities with environmental parameters.

	**All**		**Site B1**		**Site B2-A**		**Site B2-B**		**Site B3**	
**Parameter**	***R*^2^**	***p*-value**	***R*^2^**	***p*-value**	***R*^2^**	***p*-value**	***R*^2^**	***p*-value**	***R*^2^**	***p*-value**
pH	0.025	0.001^***^	0.037	0.078°						
Moisture	0.016	0.001^***^					0.043	0.006^**^	0.035	0.013^*^
TN	0.026	0.001^***^	0.043	0.017^*^					0.036	0.006^**^
TOC	0.025	0.001^***^	0.037	0.085°						
C:N ratio	0.015	0.001^***^								
Sampling site	0.116	0.001^***^								
Sampling date	0.011	0.005^**^	0.046	0.014^*^			0.041	0.008^**^	0.045	0.001^***^
Sampling season	0.027	0.003^**^			0.112	0.002^**^	0.101	0.004^**^	0.112	0.001^***^
VLP	0.020	0.001^***^								
Temperature D							0.038	0.017^*^	0.037	0.007^**^
Temperature 5D					0.033	0.073°	0.040	0.010^**^	0.035	0.012^*^
Temperature 10D					0.034	0.055°	0.037	0.025^*^	0.035	0.009^**^
Temperature M					0.033	0.065°	0.037	0.021^*^	0.034	0.025^*^
Rainfall D							0.041	0.015^*^	0.038	0.006^**^
Rainfall 5D	0.011	0.002^**^			0.036	0.023^*^	0.038	0.021^*^	0.036	0.012^*^
Rainfall 10D							0.040	0.008^**^	0.040	0.004^**^
Rainfall M							0.034	0.050^*^		

TN, total nitrogen; TOC, total organic carbon; C:N, carbon to nitrogen ratio; VLP, virus like particles; D, sampling day; 5 D, 5 days before sampling; 10 D, 10 days before sampling; M, 4 weeks before sampling.

*** ≤ 0.001,

** ≤ 0.01,

* ≤ 0.05,

° *≤ 0.1. Rho values with 0.1 > p > 0.05 are printed in gray*.

The composition of the viral communities from different samples was quite dissimilar based on Jaccard index (Table 3). Even within the individual sampling sites the mean Jaccard index was rather high with values between 0.84 and 0.90, showing that the viral communities varied considerably over time even at the same location. Noticeably, even the soil replicates of one sampling time and location were quite dissimilar (lowest Jaccard index around 0.61), indicating a high spatial and temporal heterogeneity of the viral community.

**Table 3:** Dissimilarity indices using Jaccard index from the viral community profiles.

**Sampling site**	**Minimum**	**Mean**	**Maximum**
All	0.505	0.917	1
B1	0.710	0.902	1
B2-A	0.623	0.882	1
B2-B	0.620	0.838	1
B3	0.611	0.891	1

*The closer the value is to 1 the more dissimilar are the samples. Smaller values indicate more similar samples*.

## Discussion

### Extraction efficiencies

An efficient and reliable VLP extraction method is a prerequisite for obtaining quantitative data about viruses and also for sufficient VLPs to subsequently perform molecular biological analysis steps. Among the 32 tested combinations of buffers and mechanical methods for VLP extraction, the most efficient method for the Hainich soil was 1× SM buffer in combination with vortexing, followed by diluted SM buffer and sonication or vortexing. We found that the most commonly used buffer, PC (Williamson et al., 2003, 2005, 2012, 2013; Kim et al., 2008; Wommack et al., 2009), was mostly incompatible with epifluorescence microscopy for our soil sample. Problems with enumeration of extracted viruses were previously reported for buffers containing beef extract and glycine (Williamson et al., 2003, 2005), whereas PC seemed to be a good eluent for viruses. In the present study, we could quantify VLP extracted with PC buffer (both concentrations) only when combined with a vortexing step. However, the counts were quite low compared to the other extraction methods. SP buffer, which seemed to work well in other studies (Williamson et al., 2003, 2005), resulted in intermediate to low extraction efficiencies, depending on the mechanical method used. Protocols with the diluted SP buffer performed similarly poor as the combinations with PC buffer. In contrast to the before-mentioned studies, Thurber and colleagues recommended the use of PBS or SM buffer for the generation of viral metagenomes from soil samples (Thurber et al., 2009). This is well in accordance with our results and also a good indicator that the extracted VLP can be used for further analysis by PCR- or sequencing-based approaches.

Vortexing was the only mechanical treatment which resulted in countable VLP extracts with all buffers, and was the most effective treatment in combination with 1× SM buffer and with 1× PBS. Vortexing and sonication are the most efficient mechanical methods published so far for the extraction of viruses from soil (Williamson et al., 2003, 2005, 2012, 2013; Kim et al., 2008; Wommack et al., 2009), which is in agreement with our results.

There are only few studies that actually compared different virus extraction methods for soil samples. Williamson et al. found in several individual studies, that PC buffer was most effective for most of the samples tested (Williamson et al., 2003, 2005, 2013). For clay soil samples potassium deoxycholate buffer showed a higher efficiency in extracting viruses (Williamson et al., 2013). However, the soil types Williamson and colleagues used for their studies (mainly Emporia complex, loamy sand, sandy loam, silt loam, and clay loam) were different from the one we used (rendzic leptosol), which is probably the main reason we found other buffers to be effective. These results show that the extraction method needs to be adjusted to the specific soil type prior to the actual study as the soil properties strongly influence the extraction efficiency. In the present study we only evaluated the extraction efficiency on one soil sample from one sampling site as the soil type of the other sampling sites was the same or very similar. We cannot exclude that the differing pH values may have an effect on the extraction efficiencies; pH influences the charge of viruses and soil particles, and may thus have an impact on the binding force between them (Burge and Enkiri, 1978; Taylor et al., 1981; Kimura et al., 2008).

Alike for the bacterial counterparts in soil (Philippot et al., 2012; Karpouzas et al., 2014), there is clearly a need for standardization of protocols in soil virus ecology. Developing standard protocols in soil virology would allow harmonizing datasets, performing cross-study comparisons and for integrating soil viruses into a regulatory framework.

### Virus abundance

Monitoring the VLP abundance over 1 year revealed a mean count of 1 × 10^9^ VLP g^−1^ soil (dry weight). This is well in accordance with previous studies, where the extractable amount of viruses was between 10^7^ and 10^9^ VLP g^−1^ soil (Williamson et al., 2003, 2005; Swanson et al., 2009; Amossé et al., 2013; Reavy et al., 2015; Srinivasiah et al., 2015). The standard deviation was lowest in site B3 (cropland), which is probably due to the managing of the soil (tillage), leading to a more homogeneous soil structure than at the other sampling sites. Williamson et al. found that forest soils have higher VLP counts than agricultural soils, mainly due to the higher water and soil organic matter content (Williamson et al., 2005). It has been shown that organic fertilization increased viral abundance up to 12-fold (Chen et al., 2014; Doan et al., 2014). This could be an explanation for the higher counts at the pasture site compared to the forest sites, as there were cows grazing several times each year. The dung of the cows may fertilize the soil and thus lead to higher microbial activity. Additionally, the sampling site was close to the forest line, which may alleviate the higher temperatures in summer, leading to a longer persistence of the viruses (Hurst et al., 1980; Straub et al., 1993). The soil moisture of the pasture site was similarly high as in the forest sampling sites (Figure 4) which also favors virus survival (Straub et al., 1993; Williamson et al., 2005, 2013), leading to higher VLP counts. Acidic conditions, (e.g., at site B1) may be also responsible for lower virus abundances; low pH values have been shown to affect viruses and their activity (Sykes and Williams, 1978; Sykes et al., 1981; Chen et al., 2014), and to favor virus adsorption to the soil matrix which results in lower extraction efficiency (Goyal and Gerba, 1979; Kapuscinski and Mitchell, 1980; Sykes et al., 1981).

We found significant positive correlations of VLP counts with pH for the complete dataset, indicating that with increasing pH from 3.5 to around 5 the virus abundance also increases. A pH around 5 seems to be optimal for the Hainich viral community (Supplementary Figure 2); VLP counts decreased at higher pH values. This is also supported by the correlation factors, which are positive for B1 (although not significantly) and B2-A, but negative for B3.

Our data also confirm reports that higher nitrogen and carbon contents lead to increased virus production (Williamson et al., 2005). The availability of nutrients is an important factor as it affects the nutritional state of the hosts. Viral infection of starved hosts often leads to delayed virus reproduction, as the viral genome cannot directly be reproduced and just remains in the host cell (pseudolysogeny; Miller and Day, 2008; Baess, 2009). When the metabolic state of the host is more favorable, the viral genome can integrate in the host genome (lysogenic cycle) or is directly replicated (lytic cycle) and the virus is reproduced (Kimura et al., 2008; Miller and Day, 2008). An increase in lytic infections with increasing carbon and nitrogen availability would thus lead to higher virus abundances.

Negative correlations between rainfall and virus abundances (as at B1, B2-B, and B3) may indicate that the rainfall at first dilutes and probably mobilizes and flushes out a certain amount of the viruses (Hewson et al., 2012; Williamson et al., 2014), resulting in lower virus counts. For site B2-A, however, the trees probably shield the ground from excessive wash off events, allowing only a small fraction of the rain to fall to the ground which then may increase the soil moisture and subsequently increase microbial activity leading to higher virus release. At location B1, which is also a forest site, this shielding effect could not be observed. This may be due to the trees standing not as close and site B1 being at the top of the hill and next to an open (pasture) area, which probably leads to a higher amount of rainfall reaching the ground with subsequent wash-off of microbes.

Another environmental parameter significantly correlated with virus abundances was air temperature. This correlation was negative for sites B2-B and B3, which experience a much higher evaporation at higher temperatures than the forest site B2-A. A higher evaporation leads to virus inactivation and even irreversible binding to the soil (Yeager and O'Brien, 1979); consequently the virus abundance drops. At the forest sites however, the soil stays moist for a longer time, even when the temperatures are high. This moist and warm condition may even lead to increased microbial activity, which contributes to virus release and thus higher virus abundance.

Soil moisture was only significantly correlated to viral abundance at site B2-B. Williamson et al. found a significant positive correlation of soil moisture with viral abundances (Williamson et al., 2005), but they compared different soils rather than soil moisture influences at one site. Since soil moisture influences the bacterial community (Zhang et al., 2013), and thus the potential host, it is reasonable to assume effects on the viruses as well.

The ratio of carbon to nitrogen is negatively correlated to the VLP counts for site B2-B and also for the complete dataset, but with much lower correlation strength. There are similar findings for the bacterial community, which is also negatively influenced by the C:N ratio (Zhang et al., 2013; Freedman and Zak, 2015; Delgado-Baquerizo et al., 2016).

At sites B2-A, B2-B, and B3 clear temporal and seasonal changes of VLP abundance can be seen (Figure 3). However, in order to confirm seasonal effects, further sampling across several years would be required. The increase in VLP in summer at site B2-A is mainly related to changes in temperature, rainfall, nitrogen content and soil pH as indicated by Spearman correlation (Table 1) and regression analysis. At sites B2-B and B3 the VLP counts decreased in summer which we attribute mainly to air temperature and rainfall, and which is also supported by Spearman correlation and regression analysis. Overall, the factors temperature and rainfall had an important influence on virus abundance and probably also impacted other factors such as pH. Whether temperature and rainfall is positively or negatively correlated to the VLP abundance is, based on our research, likely affected by the shielding effect of trees.

### Modified RAPD-PCR analysis (fRAPD)

The use of the modified fingerprinting method fRAPD applying fluorescence-labeled primers and a capillary sequencing machine proved to be highly reliable, time-saving and reproducible. In preliminary experiments we used pre-cast gels (see Supplementary Experimental Procedures for more details) as these seemed to be the most reliable and reproducible in terms of thickness and concentration. We found very low similarities of around 40–55% when replicate samples were run on two gels. Srinivasiah and colleagues also observed a drop in similarity when analyzing the same sample on different gels (Srinivasiah et al., 2013). The reliability and reproducibility of detecting and assigning the bands is presumably the most critical factor in the gel-based analysis. When using a capillary sequencing machine, detection and sizing of fragments is not an issue anymore as the machine detects the signal with much higher sensitivity and also with a higher throughput than in a gel-based approach. With the mean similarity of around 97% in replicate samples, fRAPD analysis on a capillary sequencing machine proved to be a reliable, highly reproducible, cost-effective and fast method for time-series, just as a T-RFLP analysis for microbial marker genes (Osborn et al., 2000; Schulz et al., 2012; Giebler et al., 2013).

### Viral community fRAPD profiles

The resulting fRAPD fingerprints of the viral communities correlated significantly to all soil specific parameters with the sampling site being the most important factor in shaping the viral community. This seems reasonable as the differences in factors such as pH and nitrogen content that distinguished the sampling sites are known to influence also the microbial community (Kennedy et al., 2004; Lauber et al., 2008; Fierer et al., 2012). Srinivasiah and colleagues showed that the soil viral community changed along with environmental factors, which also influence bacterial communities (Srinivasiah et al., 2013, 2015).

Additionally, sampling season and date, the amount of VLP and rainfall 5 days before sampling were important factors governing viral community changes. These changes are likely due to shifts in host communities, which are known to change over time (Smit et al., 2001; Griffiths et al., 2003), especially during growth and declining phases (Simek et al., 2001; Yannarell and Triplett, 2005). The correlation to the amount of VLP seems reasonable, as it is unlikely that all virus groups simultaneously increase or decrease in abundance. Based on their physicochemical properties and on host availability some viruses are much faster inactivated than others (Dowd et al., 1998; Jin et al., 2000).

Rainfall could have two main effects for soil viral communities: (i) Intense rainfall may lead to virus removal by flushing viruses out of the soil as discussed above. As different viruses have different physical properties such as surface charge or hydrophobicity, they are also flushed out at different rates (Ghanem et al., 2016). (ii) An intermediate amount of rainfall leads to increased soil moisture, can enhance the microbial activity and influence the bacterial community composition (Zhang et al., 2013), which then probably affects also the viral production and community composition. Vegetation cover may be of major importance in this context: In particular sites having less vegetation [such as B2-B (pasture) and B3 (cropland)] seemed to be more prone to weather influences.

The Jaccard index showed a temporal and geographic heterogeneity of the viral communities. However, the lowest Jaccard index found was around 0.5, which implies that there are at least some viruses that are found at several sites and may thus be more widespread. These findings are well in accordance with previous studies showing that certain virus groups are geographically more constrained than others (Fierer et al., 2007; Desnues et al., 2008; Williamson et al., 2008), but there are also viruses that are found ubiquitously (Williamson et al., 2008).

It is also known that certain soil treatments can change the abundances of specific viral groups (Chen et al., 2014). However, there is only very limited research about viral communities with respect to land use. Srinivasiah and colleagues fingerprinted several soil types and showed that the viral communities are somewhat distant in different cropping practices (Srinivasiah et al., 2013). It was also observed that the morphological diversity in forest soils was more diverse than in agricultural soils (Williamson et al., 2005). Our data provide some first indication that land use may have a strong influence on the viral community structure. However, in order to validate and confirm land use as a relevant factor shaping viral communities, future studies with more land use replicates are required.

## Conclusion

To our knowledge this is the first time-series study over 1 year that analyzes both the viral abundance and community structure and puts these data into the context of several environmental parameters. Seasonal shifts in VLP abundances could be best explained by temperature, rainfall, and soil specific factors such as nitrogen content and pH. However, the effects of rainfall and temperature were inversed at the forested site B2-A, implying an effect of forest vegetation.

We presented an optimized fingerprinting technique, called fRAPD, for the routine analysis of viral community structure which is highly suitable for fast large scale analyses of viral communities without the time consuming and error-prone gel-based analysis that was necessary before. We found that the viral community was temporally highly dynamic; moreover, the most important factor in shaping the viral community seemed to be sampling site. We further envisage that in future studies fRAPD of viral communities may serve as a pre-screening of multiple samples to even detect short-time changes, before applying the viromics tool box for a more detailed description.

## Author contributions

AnN, AC, and HH designed the study. AnN and AlN performed the field work. AnN performed laboratory work and analyzed the data. AlN performed the soil parameter analysis. AC, LW, and HH provided consultation for the work. All authors contributed significantly to the preparation of the manuscript and approve its submission.

### Conflict of interest statement

The authors declare that the research was conducted in the absence of any commercial or financial relationships that could be construed as a potential conflict of interest. The reviewer CH and handling Editor declared their shared affiliation.
