# Supplementary material for: Soil Viral Communities Vary Temporally and along a Land Use Transect as Revealed by Virus-Like Particle Counting and a Modified Community Fingerprinting Approach (fRAPD)
Source: Front Microbiol. 2017 Oct 10;8:1975. doi: 10.3389/fmicb.2017.01975 (PMC5641378; doi:10.3389/fmicb.2017.01975)
Supplement: Supplementary file 1 [file DataSheet1.pdf]

Supplementary Material

Soil viral communities vary temporally and along a land use transect as revealed by virus-like particle counting and a modified community fingerprinting approach (fRAPD)

Anja Narr, Ali Nawaz, Lukas Y. Wick, Hauke Harms, Antonis Chatzinotas*

*** Correspondence:** Antonis Chatzinotas: antonis.chatzinotas@ufz.de

1 Supplementary Materials and Methods

1.1 Reference plasmid pCR 2.1 and M13 PCR

The reference plasmid for the DNase treatment was produced using an Escherichia coli DH5α carrying the empty, self-ligated plasmid pCR 2.1 (TA Cloning® Kit, Thermo Fisher Scientific, Waltham, USA) grown on LB agar containing 50 µg/mL kanamycin. The cells were grown in liquid media overnight at 37 °C. The plasmid was extracted from the cells using the Omega E.Z.N.A.® Plasmid Mini Kit (VWR Omega, Darmstadt, Germany) according to the manufacturer's instruction. The plasmid was linearized using the restriction enzyme XcmI (New England Biolabs, Ipswich, MA, USA) at 37 °C for 1 h.

As a reference for DNase digestion, 1 µL of 0.1 µg/µL plasmid solution was added to each VLP sample (approx. 500 µL). Before the first and after each following DNase treatments the presence of the plasmid was checked using PCR. Each reaction contained 5 µL 2× Taq PCR Mastermix (Qiagen, Venlo, Netherlands), 5 pmol primer M13 forward, 5 pmol primer M13 reverse, 8 µg BSA (New England Biolabs, Ipswich, MA, USA), 1 µL VLP extract and water up to 10 µL. The cycling conditions were as follows: initial denaturation at 95 °C for 5 min, 30 cycles of (i) 95 °C for 1 min, (ii) 55 °C for 1 min and (iii) 72 °C for 1 min, final elongation was at 72 °C for 10 min followed by cool down to 8 °C. The samples were checked on a 1.5% agarose gel (LE Agarose, Biozym, Hessisch Oldendorf, Germany) cast with 0.5× TAE-buffer (20 mM Tris(hydroxymethyl)aminomethane, 10 mM Acetic acid, 0.5 mM EDTA) run at 100 V for 25 min in 0.5× TAE-buffer. A 100 bp size standard (New England Biolabs, Ipswich, MA, USA) was used to verify the size of the PCR products. Gels were stained with ethidium bromide and visualized with a transilluminator (ChemiGenius, Syngene, UK). When an approx. 200 bp band was visible the sample was DNase treated again.

1.2 Ethanol precipitation of PCR products

For the precipitation of PCR products 2.5 volumes of pure ethanol and 0.1 volume of sodium acetate (3M, pH 5.5) were added to each sample, mixed well and incubated for 5 min at room temperature. The samples were then centrifuged for 25 min at 12,000× g at room temperature and the supernatant was removed. 300 µL 70% ethanol were added, centrifuged and the supernatant removed. The sample was air dried and stored at -20 °C until further processing.

1.3 Gel-based RAPD-PCR analysis

Eight samples of viral DNA from soil were used for RAPD-PCR with primer HCB-1 (Srinivasiah et al., 2013). PCR reactions contained 2× HotStarTaq Plus Mastermix (Qiagen, Venlo, Netherlands), 20 pmol primer (biomers.net GmbH, Ulm, Germany), 1 ng DNA and water up to 40 µL. Cycling conditions were 95 °C for 5 min, 30 cycles of (i) 94 °C for 30 sec, (ii) 52 °C for 1 min and (iii) 72 °C for 1 min, final elongation was at 72 °C for 5 min followed by cool down to 8 °C. From each PCR product 2 times 15 µL were mixed with 8 µL SERVA PRiME™ DNA-loading dye (SERVA Electrophoresis GmbH; Heidelberg, Germany) and denatured at 95°C for 5 min, resulting in two replicates per sample. The PCR products were separated on SERVAGelTM TG PRiMETM 4 - 12 % gels (SERVA Electrophoresis GmbH) in 1× TBE buffer at 150 V for 75 min. A 2-log DNA ladder (New England Biolabs, Ipswich, MA, USA) was used as size reference. Gels were removed from the cast and stained in Serva DNA Stain Clear G (SERVA Electrophoresis GmbH) for 20 min, followed by 10 min destaining in distilled water and visualizing the banding pattern with a transilluminator (ChemiGenius, Syngene, Cambridge, UK). The replicates were visually very similar.

The gel images were used for analyzing the banding pattern with GelCompar II (Version 6.5, Applied Maths NV, Sint-Martens-Latem, Belgium) according to the manual. A band-matching table was exported and used for statistical analysis using R version 3.2.3 (R Core Team, 2015). The Renkonen coefficient (Renkonen, 1938) was calculated to get the percent similarity of the replicated samples.

2 Supplementary Figures and Tables

2.1 Supplementary Figures

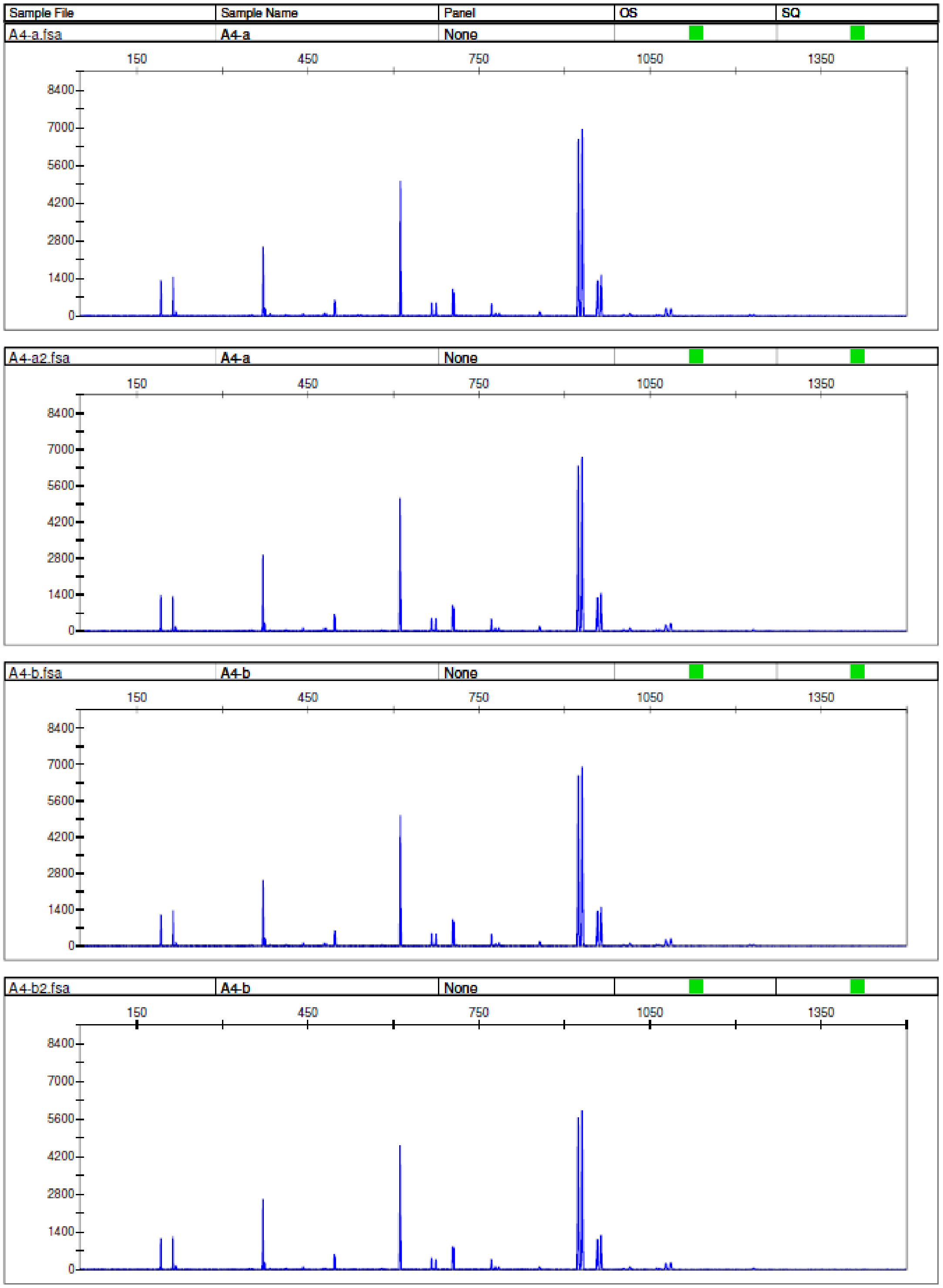

Supplementary Figure 1. Electropherogramms of replicated fRAPD measurements of the same sample.

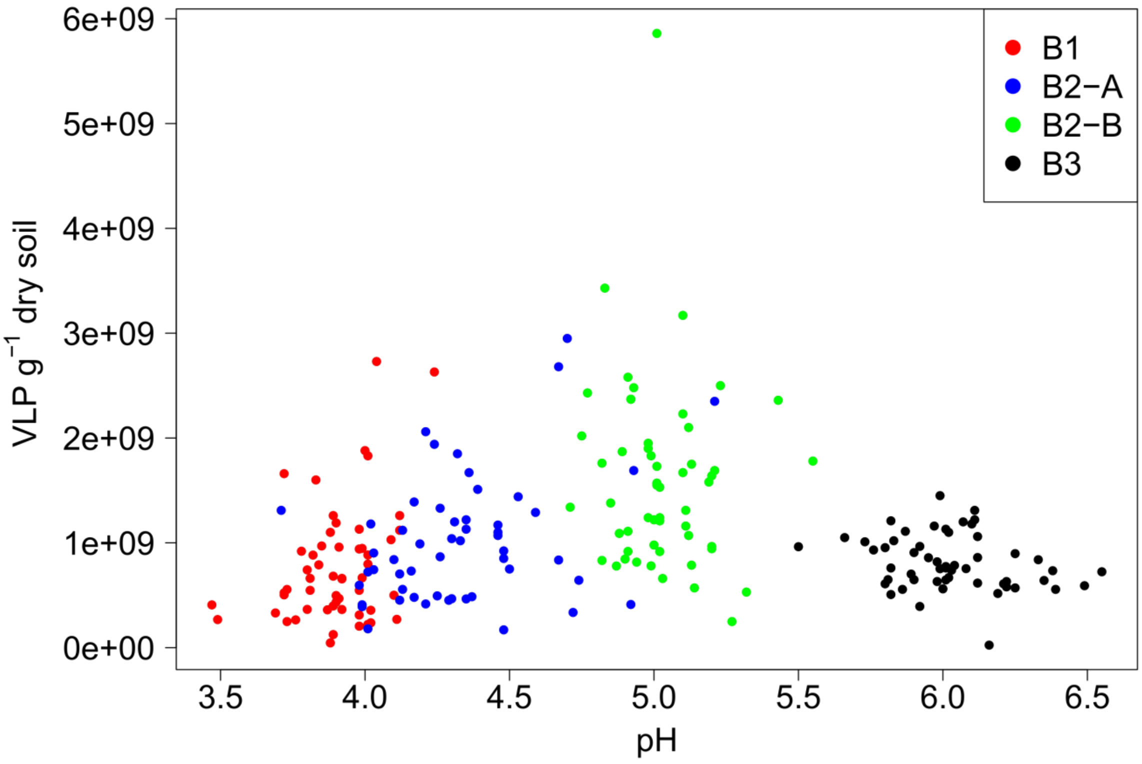

Supplementary Figure 2. Virus like particles per gram dry soil along the soil pH gradient.

2.2 Supplementary Tables

Supplementary Table 1. Overview on model parameters for modelling VLP counts from soil samples using generalized linear models with Gaussian error distribution and log-link function. AIC = Akaike Information Criterion, vif = variance inflating factor (highest vif for the predictors in the model), adjR2 = adjusted R2, Temp = temperature, RF = rainfall, TOC = total organic carbon, TN = total nitrogen content, CN = carbon to nitrogen ratio, D = sampling day, 5D = five days before sampling, 10D = ten days before sampling, M = four weeks before sampling. Values 5D, 10D and M were calculated based on the mean (temperature) or sum (rainfall) of the measurements taken on each of the five days, ten days and 4 weeks before sampling. Predictors for the different models were selected based on Spearman correlation (see Table 1), n.a. = not applicable. Asterisks mark the final model (in bold).

dataset	model	predictor(s)	AIC	vif	adj R^2
soil	null	Intercept only	9460.1	n.a.	0
full dataset	full	Temp5D, RF.D, moisture, pH, TOC, TN, CN, date	9414.1	18.6	0.220
	single	date	9457.2	n.a.	0.018
	predictor	pH	9461.4	n.a.	-0.001
		moisture	9450.7	n.a.	0.047
		TN	9444.0	n.a.	0.076
		TOC	9434.2	n.a.	0.117
		CN	9458.9	n.a.	0.010
		Temp5D	9455.7	n.a.	0.024
		RF.D	9455.3	n.a.	0.026
	two	TOC, TN	9435.4	2.0	0.116
	predictors	TOC, RF.D	9423.7	1.0	0.162
		TOC, Temp5D	9426.4	1.0	0.152
		TOC, moisture	9417.1	1.0	0.187
		TOC, date	9426.4	1.0	0.152
		TOC, CN	9434.3	1.0	0.120
		TN, RF.D	9439.0	1.0	0.101
		TN, Temp5D	9440.9	1.0	0.093
		TN, moisture	9420.6	1.0	0.174
		TN, date	9442.3	1.0	0.087
		TN, CN	9441.4	2.3	0.091
	three	**** TOC, moisture, RF.D**	**9408.0**	**1.0**	**0.224**
	predictors	TOC, moisture, Temp5D	9413.4	1.1	0.205
		TOC, moisture, date	9414.3	1.0	0.201
	four	TOC, moisture, RF.D, Temp5D	9410.0	1.9	0.220
	predictors	TOC, moisture, RF.D, date	9408.8	1.2	0.225
B1	null	Intercept only	233.9	n.a.	0
	full	Temp5D, RF.M, moisture, pH, TOC, TN, CN, date	2335.0	103.2	0.107

	one predictor	date	2331.5	n.a.	0.061
		pH	2326.3	n.a.	0.146
		moisture	2332.5	n.a.	0.043
		TN	2328.1	n.a.	0.117
		TOC	2329.0	n.a.	0.102
		CN	2335.8	n.a.	-0.017
		Temp5D	2332.5	n.a.	0.043
		RF.M	2332.4	n.a.	0.046
	two predictors	**** pH, TN**	**2326.3**	**2.1**	**0.162**
		pH, date	2327.5	1.3	0.143
		pH, TOC	2326.4	2.0	0.161
		pH, RF.M	2327.0	1.1	0.151
		pH, Temp5D	2327.4	1.2	0.145
		TN, date	2327.3	1.1	0.145
		TN, TOC	2329.4	4.2	0.112
		TN, RF.M	2328.2	1.1	0.132
		TN, Temp5D	2327.3	1.1	0.146
	three predictors	pH, TN, TOC	2327.9	4.2	0.151
		pH, TN, date	2327.4	2.4	0.160
		pH, TN, RFM	2327.4	2.2	0.158
		pH, TN, Temp5D	2327.2	2.4	0.162
B2-A	null	Intercept only	2297.6	n.a.	0
	full	TempD, RF.10D, moisture, pH, TOC, TN, CN, date	2282.2	73.5	0.346
	single predictor	date	2299.5	n.a.	-0.019
		pH	2291.8	n.a.	0.119
		moisture	2298.7	n.a.	-0.003
		TN	2287.1	n.a.	0.194
		TOC	2292.2	n.a.	0.112
		CN	2299.1	n.a.	-0.012
		TempD	2293.0	n.a.	0.098
		RF.10D	2296.0	n.a.	0.047
	two predictors	TN, pH	2285.7	1.4	0.229
		TN, TOC	2288.0	1.8	0.194
		TN, TempD	2280.5	1.0	0.301
		TN, RF.10D	2275.6	1.2	0.363
		pH, TOC	2290.0	1.5	0.163
		pH, TempD	2285.8	1.0	0.227
		pH, RF.10D	2286.7	1.0	0.214

	three predictors	TN, RF.10D, TempD	2277.5	2.5	0.350
		**** TN, RF.10D, pH**	**2274.2**	**1.7**	**0.390**
		TN, RF.10D, TOC	2277.3	2.1	0.354
	four predictors	TN, RF.10D, pH, TOC	2276.2	2.5	0.377
		TN, RF.10D, pH, TempD	2275.7	2.3	0.383
B2-B	null	Intercept only	2426.1	n.a.	0
	full	Temp5D, RF.D, moisture, pH, TOC, TN, CN, date	2429.7	29.7	0.062
	one predictor	date	2424.5	n.a.	0.044
		pH	2427.6	n.a.	-0.010
		moisture	2421.9	n.a.	0.090
		TN	2424.7	n.a.	0.041
		TOC	2428.1	n.a.	-0.019
		CN	2423.0	n.a.	0.071
		Temp5D	2419.1	n.a.	0.135
		RF.D	2424.0	n.a.	0.054
	two predictors	**** Temp5D, CN**	**2419.4**	**1.1**	**0.144**
		Temp5D, moisture	2420.1	1.7	0.134
		Temp5D, date	2421.1	1.5	0.118
		Temp5D, RF.D	2421.0	1.7	0.119
		Temp5D, TN	2420.4	1.2	0.129
		CN, moisture	2421.6	1.1	0.109
		CN, date	2421.8	1.0	0.106
		CN, RF.D	2422.8	1.1	0.090
		CN, TN	2425.0	1.9	0.053
	three predictors	Temp5D, CN, moisture	2420.7	1.6	0.138
		Temp5D, CN, RF.D	2421.4	1.7	0.127
		Temp5D, CN, TN	2421.4	1.9	0.127
		Temp5D, CN, date	2421.3	1.8	0.129
B3	null	Intercept only	2290.6	n.a.	-2.220
	full	TempD, RF.D, moisture, pH, TOC, TN, CN, date	2273.5	16.6	0.356
	one predictor	date	2287.1	n.a.	0.078
		pH	2289.0	n.a.	0.046
		moisture	2291.8	n.a.	-0.005
		TN	2289.1	n.a.	0.044
		TOC	2291.1	n.a.	0.009
		CN	2291.9	n.a.	-0.006
		**** TempD**	**2263.8**	**n.a.**	**0.396**
		RF.D	2289.8	n.a.	0.032
	two	TempD, date	2265.5	1.4	0.388

	predictors	TempD, pH	2265.7	1.1	0.386
		TempD, TN	2265.8	1.3	0.385
		TempD, RF.D	2265.5	1.4	0.389

3 Supplementary References

R Core Team (2015). *R: A language and environment for statistical computing.* Vienna, Austria: R Foundation for Statistical Computing. Available at: https://www.R-project.org/.

Renkonen, O. (1938). Statisch-ökologische Untersuchungen über die terrestrische Käferwelt der finnischen Bruchmoore. *Ann Zool Soc Bot Fenn Vanamo* 6, 1–231.

Srinivasiah, S., Lovett, J., Polson, S., Bhavsar, J., Ghosh, D., Roy, K., et al. (2013). Direct assessment of viral diversity in soils by random PCR amplification of polymorphic DNA. *Appl. Environ. Microbiol.* 79, 5450–5457. doi:10.1128/AEM.00268-13.
